# Effect of aquatic exercise on blood pressure and related physiological indicators in hypertensive patients: a systematic meta-analysis of randomized controlled trials

**DOI:** 10.3389/fphys.2026.1876479

**Published:** 2026-07-14

**Authors:** Pengli Chang, Haoyu Wang, Jierui Zhang, Yiming Zhang, Zhihao Qi, Xianyang Xin

**Affiliations:** 1 Department of General Education, Shandong First Medical University & Shandong Academy of Medical Sciences, Jinan, China; 2 Capital University of Physical Education and Sports, Beijing, China

**Keywords:** aquatic exercise, blood pressure, hypertension, meta-analysis, randomized controlled trials

## Abstract

**Background:**

Hypertension remains one of the most prevalent health risks globally. Aquatic exercise is considered an important non-pharmacological intervention for hypertension; however, its effectiveness in managing blood pressure in hypertensive patients remains controversial. The aim of this study was to systematically evaluate and conduct a meta-analysis to assess the effects of aquatic exercise on blood pressure and related parameters in individuals with hypertension.

**Methods:**

This study followed the PRISMA and Cochrane methodological guidelines. A systematic search was conducted across PubMed, Embase, Web of Science, and Cochrane Library databases. Statistical analyses and funnel plots were generated using R software for the included studies. A total of nine randomized controlled trials (RCTs), contributing up to 12 intervention-control comparisons, were included in the final analysis. Random-effects models were applied to pool effect sizes, and publication bias was assessed.

**Results:**

Nine RCTs involving 421 participants were included, contributing up to 12 intervention-control comparisons to the quantitative synthesis. Aquatic exercise significantly reduced systolic blood pressure (MD = -10.68 mmHg, 95% CI: -13.81, -7.54, *P* < 0.0001; *I*² = 17.0%) and diastolic blood pressure (MD = -5.85 mmHg, 95% CI: -8.00, -3.69, *P* < 0.0001; *I*² = 0%). However, no statistically significant improvements were found in heart rate (MD = -1.99 bpm, 95% CI: -6.47, 2.48, *P* = 0.3824; *I*² = 38.8%) or pulse wave velocity (MD = -0.68 m/s, 95% CI: -2.22 to 0.87, *P* = 0.3913; *I²* = 65.5%) (*P* > 0.05). Thus, aquatic exercise did not significantly improve arterial stiffness in hypertensive patients.

**Conclusion:**

Aquatic exercise significantly reduces systolic and diastolic blood pressure in hypertensive individuals. However, its effects on heart rate and pulse wave velocity are not statistically significant, indicating that it does not have a substantial impact on arterial stiffness in hypertensive patients. Further high-quality research is needed to confirm these findings.

## 1 Introduction

Hypertension has become one of the most significant public health challenges globally, affecting over one billion people worldwide, with numbers continuously rising over the past three decades. As a major determinant of cardiovascular morbidity and mortality, hypertension has had a substantial impact on the global burden of cardiovascular diseases (Global Report on Hypertension: The Race Against a Silent Killer; NCD Risk Factor Collaboration (NCD-RisC), 2021). Research indicates that a reduction of approximately 10 mmHg in systolic blood pressure can lower the risk of cardiovascular events by around 25% and reduce the risk of stroke by about 30% (Brunström and Carlberg, 2018; Li and Li, 2019). Therefore, effectively controlling resting blood pressure is crucial for reducing disease burden and improving public health on a global scale.

In addition to pharmacological treatments, lifestyle interventions are considered key components in the management of hypertension (Fu et al., 2020; Verma et al., 2021; Kodela et al., 2023). Lifestyle modifications are closely related to health management in hypertensive patients, and numerous meta-analyses have shown that lifestyle changes can reduce blood pressure (Kelley, 1999; Kelley and Sharpe Kelley, 2001; Kelley et al., 2001; Xin et al., 2001; Whelton et al., 2002; Dickinson et al., 2006; Cornelissen et al., 2011; Cornelissen and Smart, 2013; Graudal et al., 2020). Among these non-pharmacological interventions, aquatic exercise, with its unique physical properties such as buoyancy, warm water, and hydrostatic pressure, not only offers advantages in relieving joint stress and enhancing exercise comfort but may also outperform certain high-intensity land-based exercises in terms of safety and adherence (Ws et al., 2023; Xin et al., 2024; Eichel et al., 2025). Aquatic exercise exerts its blood pressure-lowering effects through various mechanisms, primarily by improving endothelial function, reducing arterial stiffness, promoting venous return due to water’s hydrostatic pressure, thereby lowering systemic vascular resistance, and inhibiting sympathetic nervous activity (Fukuie et al., 2021; Weenink and Wingelaar, 2021). Studies have shown that regular aquatic exercise significantly reduces blood pressure in hypertensive patients. A meta-analysis based on randomized controlled trials reported that systematic aquatic exercise can reduce average resting systolic blood pressure by approximately 8.4 mmHg and diastolic blood pressure by around 3.3 mmHg (Dunlap et al., 2025).

Currently, while much evidence supports the benefits of aquatic exercise for hypertensive patients, the extent of blood pressure reduction across different studies is inconsistent. Variations in intervention methods, exercise intensity, and duration could all influence the effectiveness of the intervention. Furthermore, the inclusion criteria of previous studies, the quality of trial designs, and the lack of differentiation between various exercise modalities have, to some extent, affected the reliability of the conclusions. Although there is substantial evidence supporting the potential blood pressure-lowering effects of aquatic exercise, the sources of heterogeneity remain unclear. Given the importance of hypertension management, this study aims to systematically integrate high-quality RCT evidence through a meta-analysis, clarify the true intervention effects of aquatic exercise on resting systolic and diastolic blood pressure, as well as related physiological parameters, and explore potential influencing factors. This will provide evidence-based support for the development of exercise intervention programs and hypertension prevention strategies.

## 2 Materials and methods

This review was conducted in accordance with the reporting standards for meta-analyses (PRISMA) (B et al., 2015) and the methodological guidance of the Cochrane Handbook for Systematic Reviews of Interventions (Cochrane Handbook for Systematic Reviews of Interventions | Cochrane). The protocol was prospectively registered in the PROSPERO database (registration number: CRD420251248255).

### 2.1 Aquatic exercise and eligibility criteria

Studies were eligible if they met the following criteria based on the PICOS framework (Santos et al., 2007): Participants: Participants were clinically diagnosed with primary hypertension, with or without antihypertensive medication, without restrictions on sex or age. Hypertension was defined according to the diagnostic criteria used in the original RCTs, commonly including office systolic blood pressure ≥140 mmHg and/or diastolic blood pressure ≥90 mmHg, or current antihypertensive treatment. Studies using other internationally recognized diagnostic thresholds, such as the ACC/AHA threshold of systolic blood pressure ≥130 mmHg and/or diastolic blood pressure ≥80 mmHg, were also considered eligible. Diagnostic criteria followed internationally recognized guidelines issued by the World Health Organization (WHO), the American Heart Association (AHA), or Chinese hypertension management guidelines (Whelton et al., 2018; Joint Committee for Guideline Revision, 2019; Guideline for the Pharmacological Treatment of Hypertension in Adults, 2021). Interventions: The intervention consisted of aquatic exercise, including but not limited to swimming, aquatic walking, aquatic aerobic training, and water-based resistance training. In this review, “aquatic exercise” was defined as a structured, planned, and repeated exercise intervention performed in a water-based environment, with the primary purpose of improving physical or cardiovascular function. Therefore, swimming, aquatic walking, aquatic aerobic training, and water-based resistance training were classified under the broader category of aquatic exercise. Studies in which water-based activity was only a minor or inseparable component of a broader rehabilitation program were excluded. Comparators: Control groups received no exercise intervention or only low-intensity activities (e.g., relaxation, stretching, routine care). If both groups received additional concurrent measures (e.g., medication, dietary management), the study remained eligible as long as these conditions were equivalent across groups. Outcomes: Outcomes included at least resting systolic or diastolic blood pressure; studies reporting ambulatory blood pressure (e.g., 24-h ABPM) were also included. Study design: Study design was a randomized controlled trial (RCT) with full-text availability.

Exclusion criteria were as follows (1): Participants with normotension, hypotension, gestational hypertension, or secondary hypertension. (2) Interventions that did not clearly involve aquatic exercise or studies in which aquatic activity was only a minor component of a broader rehabilitation program. (3) Control groups that received additional structured exercise, making it impossible to isolate the effect of aquatic exercise. (4) Studies that did not report blood pressure outcomes, or that only reported heart rate, metabolic parameters, or other non–blood pressure outcomes. (5) Non-RCT designs, reviews, case reports, conference abstracts, or duplicate publications. (6) Articles not published in Chinese or English.

### 2.2 Literature search strategy

A systematic search was conducted in PubMed, the Cochrane Library, Web of Science, and Embase to identify RCTs examining the effects of aquatic exercise in hypertensive populations. The search covered studies published from 1990 to June 1, 2026. Both MeSH terms and free-text keywords were used, and reference lists of previously published reviews and meta-analyses were manually screened to identify additional eligible studies. Detailed search strategies for PubMed and Embase are provided in **Supplementary Tables 1**-**4**.

### 2.3 Study selection and data extraction

Two reviewers (PL-C, HY-W) independently screened the studies and extracted data using EndNote software, and discrepancies were resolved through discussion. If disagreement persisted, a third reviewer (YM-Z) adjudicated. After removing duplicates, titles and abstracts were screened to exclude studies clearly unrelated to the topic (e.g., non-hypertensive samples, non-aquatic interventions, non-RCT designs). Full texts of potentially eligible studies were assessed based on the predefined criteria. Extracted information included study characteristics, participant demographics, intervention details, outcome measures, study design features, and methodological quality. When outcome data were incomplete, corresponding authors were contacted for clarification. If data could not be retrieved, this was documented in the analysis. To ensure comparability across trials, we also prespecified the timing of outcome extraction. Outcome assessment timing: We extracted resting SBP/DBP (and resting HR, when available) measured at baseline and at the end of the intervention under standardized resting conditions. When trials reported the time lag after the final session, post-intervention measurements were performed ≥24 h after the last exercise bout (e.g., 24–48 h in Tanaka et al.; 48–72 h in Mohr et al.) to reduce contamination by acute exercise effects. If the lag was not reported, outcomes were treated as end-of-intervention resting measures as described in the original reports.

### 2.4 Risk of bias assessment

Risk of bias was independently assessed by two reviewers (PL-C, ZH-Q) using the Cochrane Risk of Bias tool for randomized trials (RoB 1, version 5.1.0) as described by Higgins et al. (2011). Disagreements were resolved by discussion, or by a third reviewer (YM-Z) when necessary. The assessment covered seven domains: random sequence generation, allocation concealment, blinding of participants and personnel, blinding of outcome assessment, completeness of outcome data, selective reporting, and other potential sources of bias (Higgins et al., 2011). Based on these criteria, studies were classified into three quality levels; High quality: no domains rated as high risk, with ≤3 domains rated as unclear risk; Moderate quality: one high-risk domain, or no high-risk domains but ≥4 unclear-risk domains; Low quality: all other cases (e.g., ≥2 high-risk domains or overall methodological weaknesses likely to affect validity).

### 2.5 Statistical analysis

Statistical analyses were performed using R (version 4.4.2) with the meta and metafor packages (Viechtbauer, 2010). To minimize the influence of baseline differences, effect sizes were calculated using mean changes and corresponding standard deviations (SDs) before and after the intervention. Continuous outcomes were expressed as mean differences (MDs) with 95% confidence intervals (95% CIs). When outcome units were inconsistent across studies, standardized mean differences (SMDs) were used. Handling of multi-arm trials: Some trials included multiple eligible aquatic intervention arms. To avoid double counting participants in shared control groups, each intervention arm was treated as an independent comparison, and the sample size of the shared control group was split equally across comparisons, while means and standard deviations were kept unchanged, following Cochrane recommendations (Cochrane).

Heterogeneity was assessed using the I²statistic, with thresholds of <50% indicating low heterogeneity, 50–75% indicating moderate heterogeneity, and >75% indicating high heterogeneity (Higgins et al., 2003). Given anticipated variation in study design, intervention modalities, and participant characteristics, a random-effects model was applied, assuming that true effect sizes followed a normal distribution. Because the number of available trials was limited, separate subgroup analyses by aquatic exercise modality were not feasible. Therefore, these modalities were pooled under the broad category of aquatic exercise, and a random-effects model was used to account for between-study variability in exercise modality, intensity, and duration. For analyses with I² ≥ 50%, sensitivity analyses were performed by sequentially removing individual studies to evaluate the robustness of pooled estimates. All statistical tests were two-tailed, with P < 0.05 considered statistically significant.

### 2.6 Certainty of evidence assessment

The certainty of evidence for each outcome was assessed using the GRADE approach. The following five domains were considered: risk of bias, inconsistency, indirectness, imprecision, and publication bias. Because all included studies were randomized controlled trials, the initial certainty of evidence was rated as high and was downgraded when serious or very serious concerns were identified. The certainty of evidence was classified as high, moderate, low, or very low (Guyatt et al., 2008).

## 3 Results

### 3.1 Study selection process

**Figure 1:**
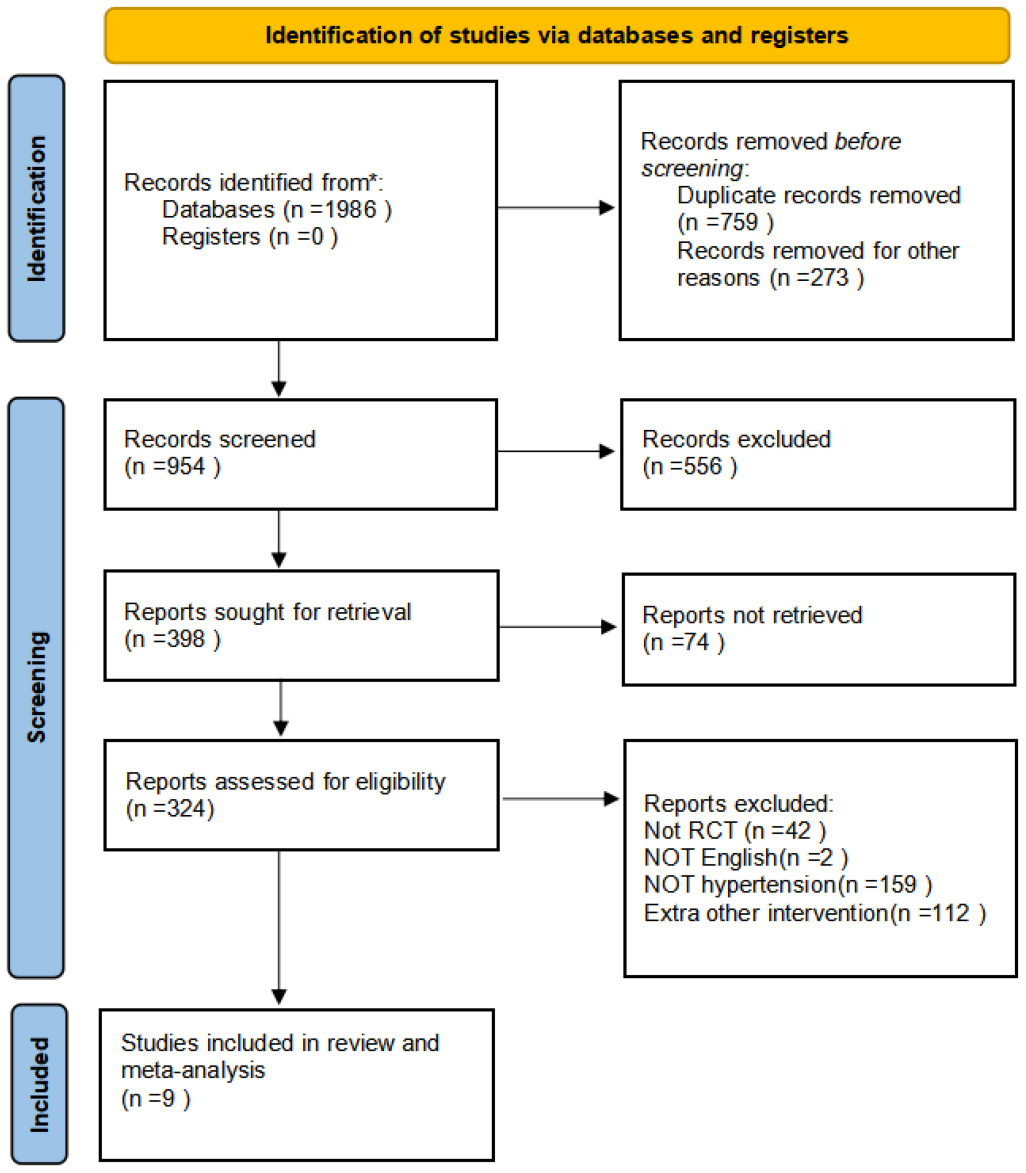
Study selection flowchart.

A total of 1986 records were identified through database searches, including PubMed (n = 222), Embase (n = 1018), the Cochrane Library (n = 170), and Web of Science (n = 576). Before screening, 759 duplicate records and 273 records removed for other reasons were excluded, leaving 954 records for title and abstract screening. After title and abstract screening, 556 records were excluded, and 398 reports entered the next stage of screening. Subsequently, 324 reports were assessed for eligibility. Full-text assessment was performed for the remaining articles, and 9 studies met all criteria and were included in the final analysis (Tanaka et al., 1997; Nualnim et al., 2012; Arca et al., 2014; Guimaraes et al., 2014; Mohr et al., 2014; Cruz et al., 2017; Wong et al., 2018; Ngomane et al., 2019; Dunlap et al., 2023). Because several trials included more than one eligible aquatic exercise arm, these nine RCTs contributed 12 intervention-control comparisons to the quantitative synthesis. In total, 421 participants were enrolled across the included trials, with 255 assigned to aquatic exercise interventions and 166 assigned to control conditions. The study selection flow is presented in **Figure 1**.

### 3.2 Characteristics of included studies

A total of 421 participants were included across the studies. Participant age ranged from 42 to 90 years, and BMI ranged from 21.9 to 35.7 kg/m². All interventions involved aquatic exercise, with water walking being the most common modality. Other forms included swimming, aquatic aerobics, and water-based stretching. Detailed study characteristics are summarized in **Table 1**.

**Table 1:** Characteristics of included studies.

First author	Region	Sample (experimental group/control group)	Intervention cycle (weeks)	Intervention frequency (times/week)	Intervention time (min)
Nualnim, 2012	America	24/19	12	3-4	30
Wong, 2018	America	52/48	20	3-4	35
Tanaka, 1997	America	12/6	10	3	60
Ngomane, 2019(1)	Brazil	15/7	12	4	30
Ngomane, 2019(2)	Brazil	15/8	12	4	30
Mohr, 2014(1)	Britain	21/10	15	3	15-25
Mohr, 2014(2)	Britain	21/10	15	3	60
Guimaraes, 2014	Brazil	16/16	12	3	60
Dunlap, 2023	Brazil	13/12	12	3	60
Cruz, 2017	Brazil	28/16	12	3	60
Arca, 2014(1)	Brazil	19/7	12	3	50
Arca, 2014(2)	Brazil	19/7	12	3	50

Rows represent intervention-control comparisons rather than separate studies. For multi-arm trials, eligible aquatic exercise arms are presented separately, and the shared control group was split across comparisons to avoid double counting. Intervention duration refers to the total intervention period in weeks; frequency refers to the number of exercise sessions per week; session duration refers to the duration of each exercise session in minutes.

### 3.3 Risk of bias assessment

**Figure 2:**
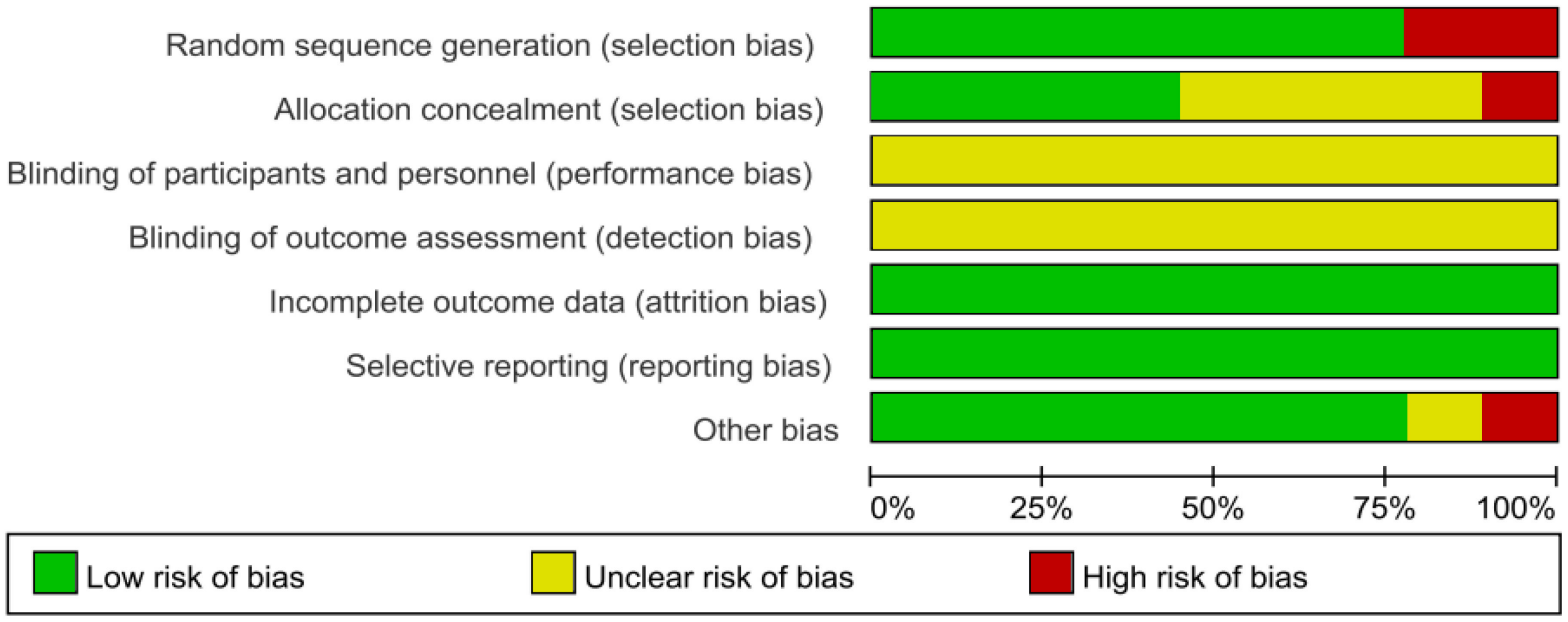
Summary of risk of bias for included studies.

**Figure 3:**
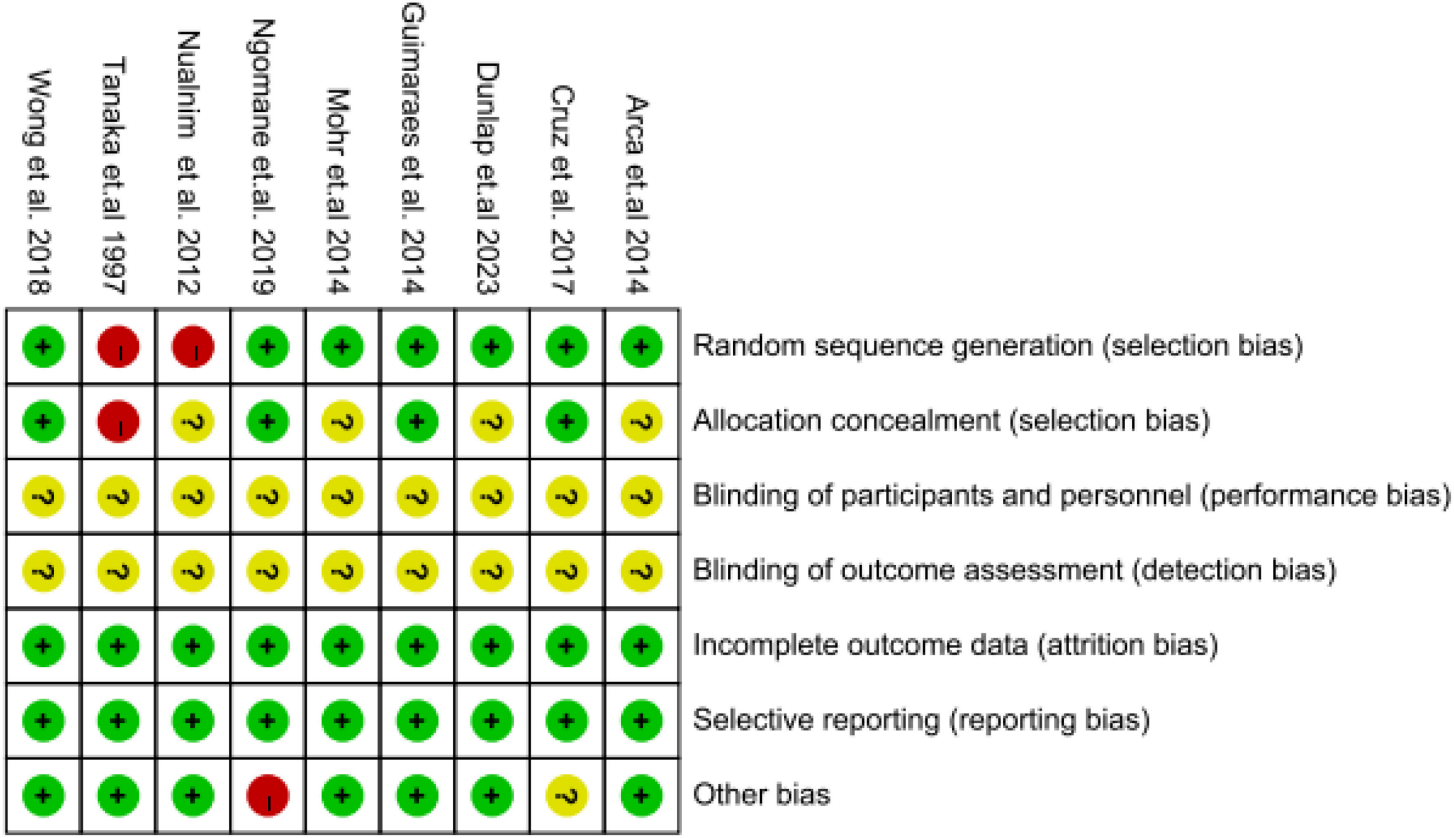
Risk of bias graph.

Risk of bias assessments for the included trials are presented in **Figures 2**, **3**. Regarding random sequence generation, 7 studies explicitly reported their randomization methods, while 2 were rated as high risk. For allocation concealment, 4 studies provided clear descriptions, 4 were unclear, and 1 was rated as high risk. Blinding of participants, personnel, and outcome assessors was rarely reported; therefore, all 9 studies were rated as “unclear risk” for these domains. For completeness of outcome data, all studies reported follow-up results and outcome measurements, suggesting an overall low risk of attrition bias. Selective reporting was not detected in any study. For other potential sources of bias, 7 studies were rated as low risk, 1 as unclear, and 1 as high risk. Combining the risk ratings across all domains, 6 studies were classified as high quality, 2 as moderate quality, and 1 as low quality.

### 3.4 Meta-analysis results

#### 3.4.1 Effect of aquatic exercise on systolic blood pressure

**Figure 4:**
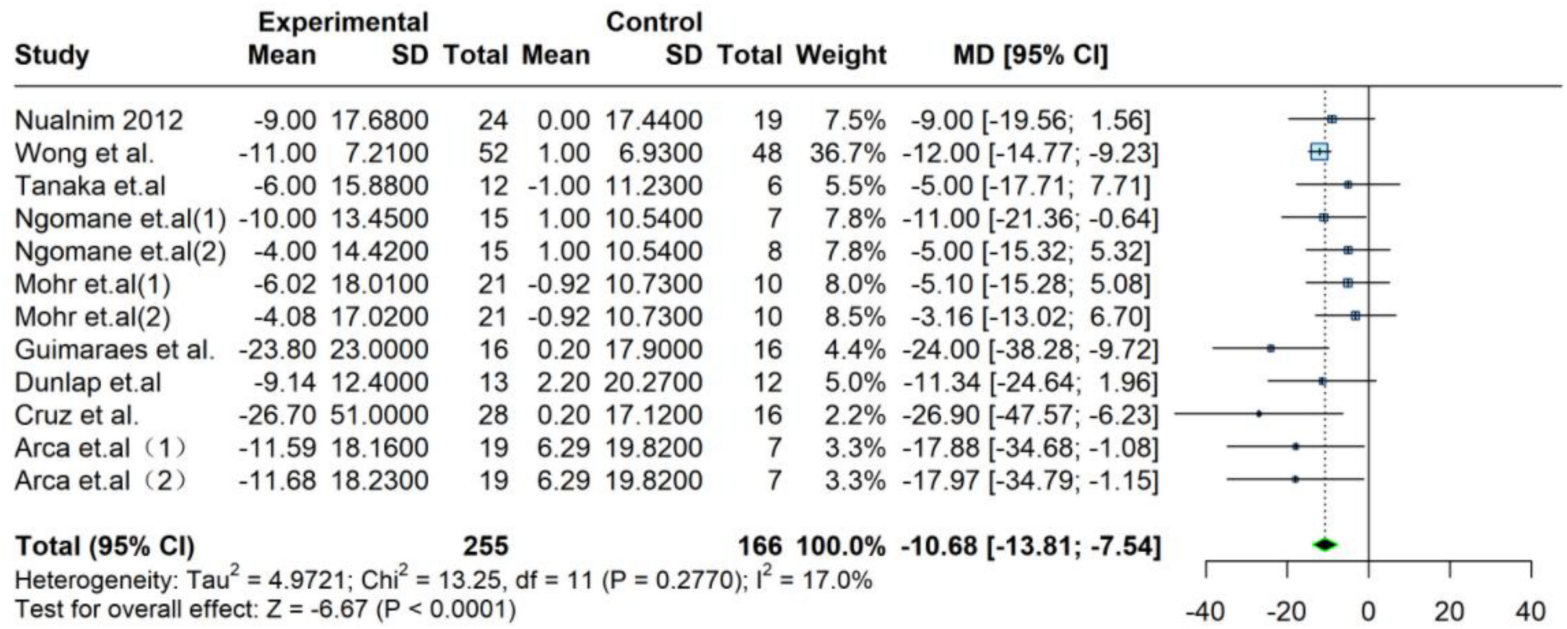
Forest plot of the effect of aquatic exercise on SBP.

Nine studies evaluating the effect of aquatic exercise on SBP were included. The meta-analysis showed low heterogeneity (*I*² = 17.0%, *P* = 0.2770) (**Figure 4**). Considering variations in study design, intervention characteristics, and participant profiles, a random-effects model was applied to estimate the pooled effect size. The pooled estimate showed a statistically significant aquatic exercise-associated change in SBP compared with control conditions (MD = -10.68 mmHg, 95% CI: -13.81 to -7.54, *P* < 0.0001). These findings suggest a potentially favorable SBP-related association in hypertensive patients.

#### 3.4.2 Effect of aquatic exercise on diastolic blood pressure

**Figure 5:**
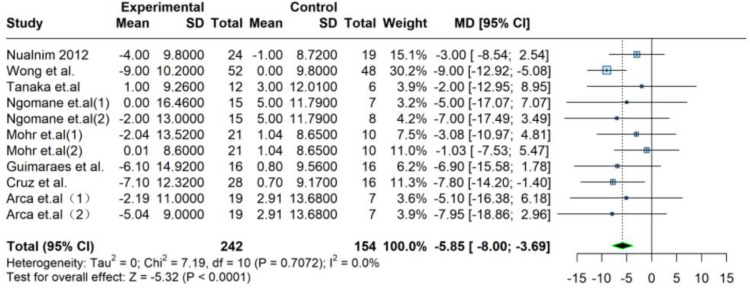
Forest plot of the effect of aquatic exercise on DBP.

Nine studies reported the effect of aquatic exercise on DBP. Heterogeneity was negligible (*I*²= 0%, *P* = 0.7072) (**Figure 5**). A random-effects model was therefore used. The pooled estimate showed a statistically significant aquatic exercise-associated change in DBP (MD = -5.85 mmHg, 95% CI: -8.00 to -3.69, *P* < 0.0001). These results suggest that aquatic exercise may be associated with favorable DBP-related changes among individuals with hypertension.

#### 3.4.3 Effect of aquatic exercise on heart rate

**Figure 6:**
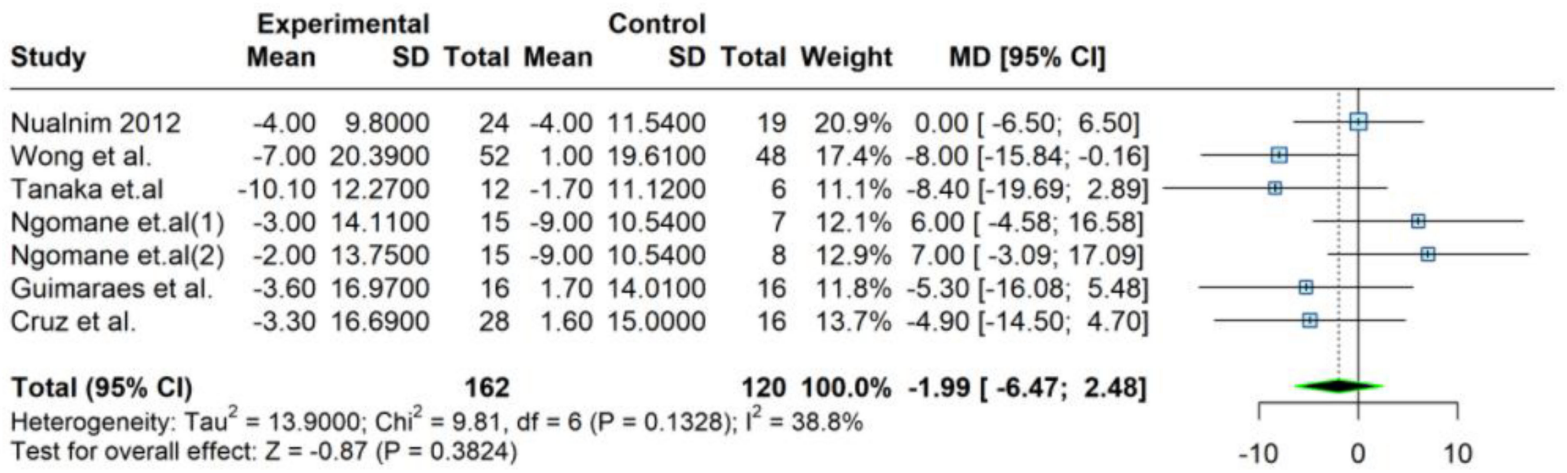
Forest plot of the effect of aquatic exercise on heart rate.

Six studies assessed the impact of aquatic exercise on resting heart rate. The meta-analysis indicated moderate heterogeneity (*I*² = 38.8%, *P* = 0.1328) (**Figure 6**). A random-effects model was used to account for methodological and population differences. The pooled estimate showed no statistically significant aquatic exercise-associated change in heart rate (MD = -1.99 bpm, 95% CI: -6.47 to 2.48, *P* = 0.3824). This suggests that the available evidence does not support a clear association between aquatic exercise and resting heart rate changes in hypertensive patients.

#### 3.4.4 Effect of aquatic exercise on pulse wave velocity

**Figure 7:**
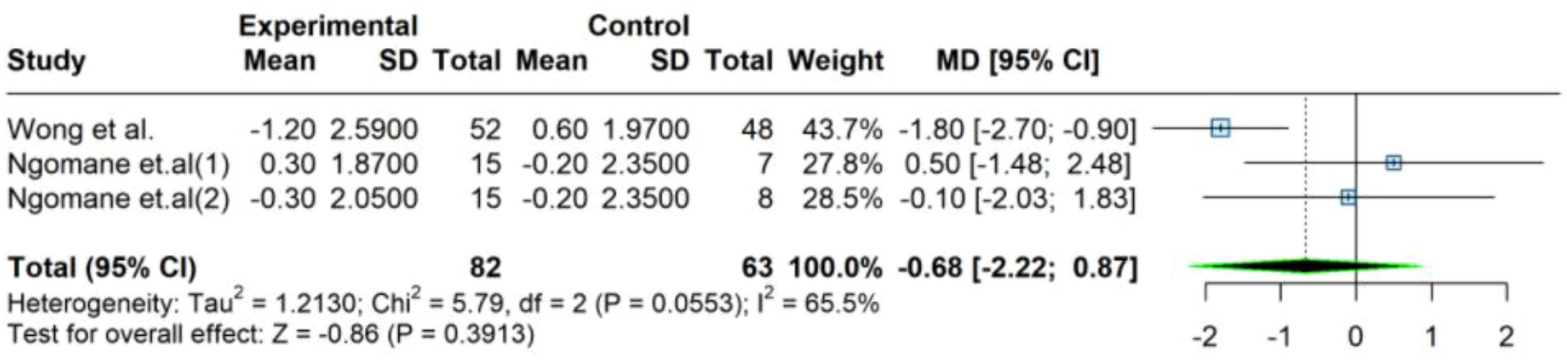
Forest plot of the effect of aquatic exercise on PWV.

Two randomized controlled trials reported PWV outcomes, contributing three comparisons to the meta-analysis (one from Wong et al. and two arms from Ngomane et al.), and substantial heterogeneity was observed (*I²* = 65.5%, *P* = 0.0553). (**Figure 7**), and a random-effects model was applied. The pooled estimate showed no statistically significant aquatic exercise-associated change in PWV (MD = -0.68 m/s, 95% CI: -2.22 to 0.87; Z = -0.86, *P* = 0.3913). These findings indicate that the current evidence does not demonstrate a clear aquatic exercise-associated change in arterial stiffness as measured by PWV.

### 3.5 Publication bias assessment

**Figure 8:**
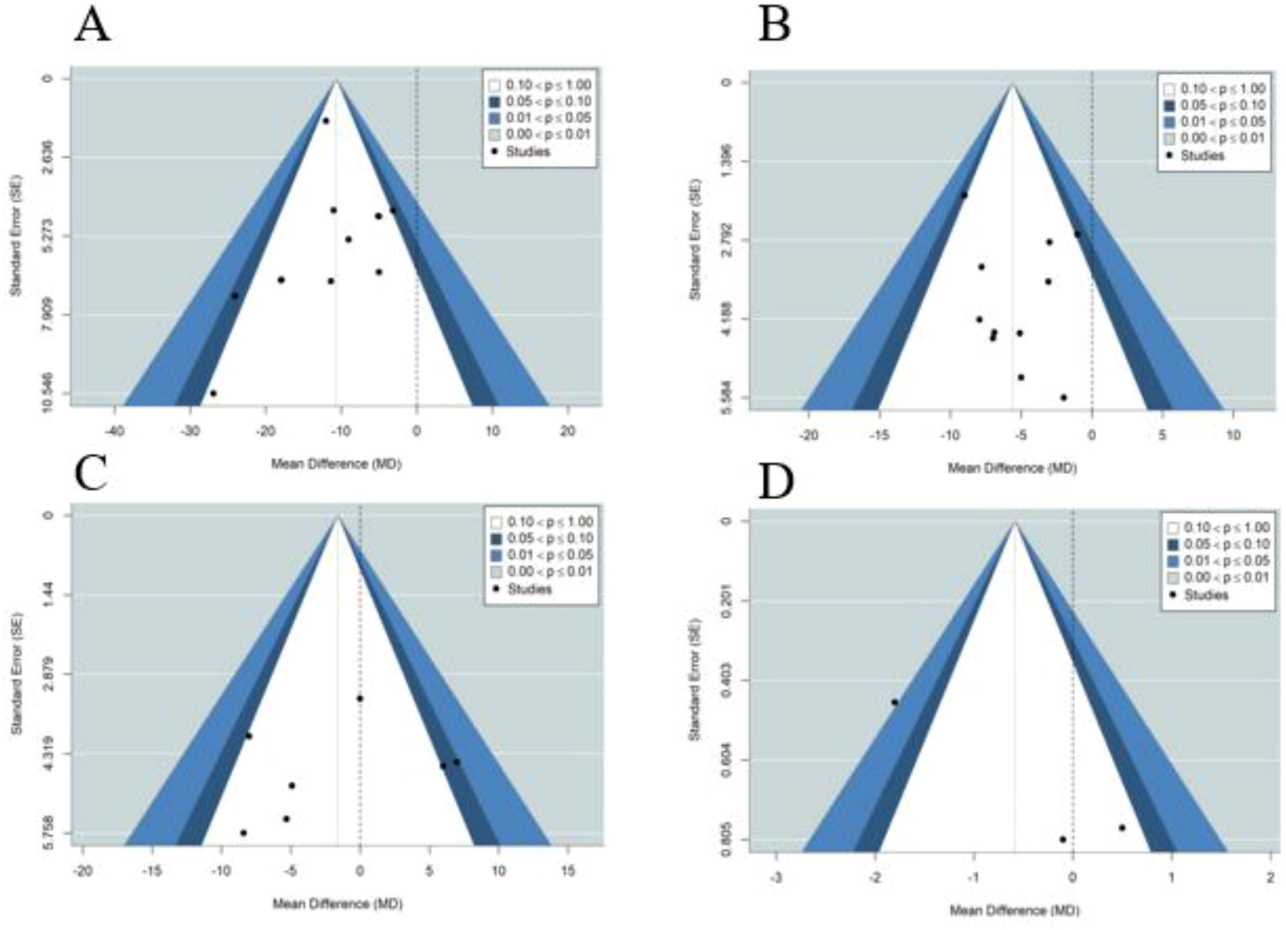
Funnel plots for SBP **(A)**, DBP **(B)**, heart rate **(C)**, and PWV **(D)**.

Publication bias was evaluated using funnel plots for the primary outcomes (**Figure 8**). The plots for SBP, DBP, and heart rate showed overall symmetry, with data points clustering near the top of the inverted funnel, suggesting a low likelihood of publication bias. However, for PWV, the limited number of included studies made it difficult to reliably assess bias. Visual inspection of the funnel plots did not suggest marked asymmetry. However, given the small number of included studies, particularly for PWV, the assessment of publication bias was limited and should be interpreted with caution.

### 3.6 Certainty of evidence

The certainty of evidence was assessed using the GRADE approach and is summarized in **Table 2**. The certainty of evidence was rated as moderate for SBP and DBP, low for heart rate, and very low for PWV.

**Table 2:** GRADE summary of findings for the certainty of evidence.

Outcome	Evidence base	Effect estimate	Risk of bias	Inconsistency	Indirectness	Imprecision	Publication bias	Certainty
SBP	9 RCTs/12 comparisons; n = 421	MD = -10.68 mmHg 95% CI: -13.81 to -7.54	Serious^a^	Not serious	Not serious	Not serious	Not serious^b^	Moderate
DBP	8 RCTs/11 comparisons; n = 396	MD = -5.85 mmHg 95% CI: -8.00 to -3.69	Serious^a^	Not serious	Not serious	Not serious	Not serious^b^	Moderate
Heart rate	6 RCTs/7 comparisons; n = 282	MD = -1.99 bpm 95% CI: -6.47 to 2.48	Serious^a^	Not serious	Not serious	Serious^c^	Not serious^b^	Low
PWV	2 RCTs/3 comparisons; n = 145	MD = -0.68 m/s 95% CI: -2.22 to 0.87	Serious^a^	Serious^d^	Not serious	Very serious^e^	Unable to assess^f^	Very low

GRADE certainty of evidence was assessed across five domains: risk of bias, inconsistency, indirectness, imprecision, and publication bias. Randomized controlled trials started at high certainty and were downgraded when serious or very serious concerns were identified. CI, confidence interval; DBP, diastolic blood pressure; MD, mean difference; PWV, pulse wave velocity; RCT, randomized controlled trial; SBP, systolic blood pressure.

^a^Downgraded by one level because some included trials had unclear or high risk in random sequence generation, allocation concealment, blinding, or other bias domains.

^b^Funnel plot assessment was limited by the small number of included studies, but no clear evidence of publication bias was identified.

^c^Downgraded by one level because the confidence interval crossed the line of no effect and included both potential benefit and no effect.

^d^Downgraded by one level because heterogeneity was moderate-to-substantial for PWV.

^e^Downgraded by two levels because PWV was based on only two RCTs and three comparisons, with a wide confidence interval crossing the line of no effect.

^f^Publication bias could not be reliably assessed for PWV because only two studies contributed to this outcome.

## 4 Discussion

Hypertension is one of the most important cardiovascular risk factors worldwide. It not only markedly increases the risk of cerebrovascular and cardiovascular events and all-cause mortality, but is also closely associated with multiple pathological mechanisms, including endothelial dysfunction and heightened sympathetic nervous system activity (Grassi et al., 2015; Vanhoutte et al., 2017). As a specific form of exercise, aquatic exercise makes use of buoyancy, resistance, and thermal effects provided by the water environment, and can lower blood pressure by improving hemodynamics and modulating neurohumoral regulation. However, some studies have reported that aquatic exercise does not significantly affect heart rate in patients with hypertension (Guimaraes et al., 2014; Cruz et al., 2017), and one trial even suggested that it may increase resting heart rate (Ngomane et al., 2019),which may be partly explained by elevated water temperature during exercise leading to an increase in heart rate.

Therefore, the evidence regarding aquatic exercise-associated blood pressure changes in hypertensive patients remains somewhat inconsistent. By conducting a systematic review and meta-analysis of the available high-quality evidence, this study aimed not only to clarify aquatic exercise-associated changes in blood pressure, but also to provide an evidence base for developing individualized exercise prescriptions and enriching non-pharmacological treatment options for hypertension.

Systolic blood pressure (SBP) and diastolic blood pressure (DBP) are key clinical indicators for evaluating cardiovascular risk in patients with hypertension. Elevated SBP is strongly associated with the incidence of myocardial infarction, heart failure, and stroke, whereas abnormal DBP is often linked to increased peripheral resistance and structural and functional changes in small arteries. Large epidemiological studies have shown that a 10 mmHg reduction in SBP can lower coronary heart disease risk by approximately 20% and stroke risk by about 30%, while a 5 mmHg reduction in DBP can also significantly decrease the incidence of adverse cardiovascular events (Lewington et al., 2002). These findings underscore the decisive role of SBP and DBP control in improving prognosis and reducing all-cause mortality.

The present meta-analysis showed that aquatic exercise-associated changes were statistically significant for both SBP and DBP in patients with hypertension, suggesting potentially favorable blood pressure-related associations. This is consistent with the findings of most randomized controlled trials and systematic reviews, and supports aquatic exercise as an effective non-pharmacological intervention. Most researchers attribute favorable aquatic exercise-associated blood pressure changes to improvements in endothelial function, reductions in sympathetic activity, enhanced parasympathetic tone, and increased nitric oxide bioavailability (Cooke and Dzau, 1997; McIntyre and Dominiczak, 1997; Pendergast et al., 2015; Vanhoutte et al., 2017). In addition, hydrostatic pressure facilitates venous return and augments cardiac output, thereby improving circulatory dynamics, which is considered another important mechanism contributing to blood pressure reduction (Pendergast et al., 2015).Beyond these general exercise-related mechanisms, immersion in water introduces several context-specific physiological stimuli that may further contribute to blood pressure reduction. First, hydrostatic pressure increases central blood volume and cardiopulmonary baroreceptor loading, which may suppress sympathetic outflow and facilitate vascular relaxation (Miwa et al., 1996). Second, thermoneutral or mildly warm water can reduce peripheral vasoconstrictor tone and improve microvascular perfusion, thereby lowering systemic vascular resistance (Boussuges, 2006). Third, buoyancy reduces weight-bearing stress and musculoskeletal strain, allowing hypertensive individuals to achieve an adequate training dose with lower mechanical load and potentially improved adherence (Kutzner et al., 2017). Collectively, these immersion-related effects may complement training-induced endothelial and autonomic adaptations and contribute to favorable end-of-intervention resting SBP/DBP profiles. In addition to dynamic aerobic and resistance training, accumulating evidence suggests that isometric exercise training (e.g., isometric handgrip) may produce clinically meaningful reductions in blood pressure (Cornelissen and Smart, 2013; Carlson et al., 2014). However, the trials included in the present review primarily evaluated aquatic dynamic exercise programs (swimming, water-based aerobic/resistance training), and isometric protocols were not a focus of these interventions. Future trials may consider examining whether incorporating isometric components into aquatic programs provides additional benefits for blood pressure control.

It should be noted that differences in exercise modality, intensity, and intervention duration across studies may lead to variability in the magnitude of blood pressure-related changes. For example, Mohr et al. (2014) reported that participants undergoing high-intensity interval swimming showed more favorable SBP and DBP changes compared with those performing moderate-intensity continuous swimming. However, this finding is not yet supported by a larger body of evidence, and the relative advantages of high-intensity interval versus moderate-intensity continuous aquatic exercise in terms of blood pressure control remain controversial and warrant further investigation.

Overall, the results of this meta-analysis highlight the potential clinical value of aquatic exercise-associated SBP and DBP changes and further support its clinical value in the management of hypertension. Future studies should integrate different exercise modes, intervention durations, and patient characteristics to identify the optimal aquatic exercise prescription and better guide exercise-based interventions for hypertensive patients.

Heart rate is an important physiological marker reflecting autonomic nervous system activity and cardiovascular regulation, and is also a key parameter in assessing cardiovascular risk in patients with hypertension. Previous studies have shown that changes in heart rate are closely related not only to exercise mode and intensity, but also to environmental factors specific to aquatic settings, such as water temperature and water depth (Barbosa et al., 2009).

The present meta-analysis suggests that, overall, aquatic exercise induces a slight reduction in resting heart rate among hypertensive patients, indicating a potentially beneficial effect on autonomic balance and cardiovascular regulation. However, there was some heterogeneity among the included studies, which may be attributable to differences in intervention protocols, participant characteristics, and environmental conditions. Nevertheless, as all included studies were randomized controlled trials and the intervention and control groups were comparable in all aspects except the exercise intervention, the overall conclusions of this analysis remain relatively robust.

It is worth noting that Ngomane et al. (2019)observed an upward trend in resting heart rate after aquatic exercise training. This may reflect differences in intervention characteristics, particularly water temperature, which can shape thermoregulatory and hemodynamic demands during training and thereby influence longer-term autonomic adaptation. For example, warmer-water immersion can lower peripheral vascular resistance and increase cardiac workload, potentially attenuating reductions in resting HR over time. Therefore, water temperature should be considered when interpreting end-of-intervention resting HR changes (Srámek et al., 2000).

The mechanisms underlying aquatic exercise-associated heart rate changes have not been fully elucidated and likely involve multiple regulatory pathways. Different exercise intensities and intervention durations appear to exert differential effects on heart rate. Moreover, individual factors (such as baseline heart rate, medication use, and exercise tolerance) and experimental conditions (such as timing of measurement and heart rate assessment methods) may also influence the results. Future research should further standardize water temperature, water depth, and intensity grading in aquatic interventions and clarify the specific regulatory effects of different exercise prescriptions on heart rate across various patient subgroups.

Arterial stiffness is a critical component in cardiovascular risk assessment among patients with hypertension, and pulse wave velocity (PWV) is widely regarded as one of the most sensitive and reliable indices of arterial elasticity and early arteriosclerosis (Townsend et al., 2015). Accelerated PWV is closely associated with reduced arterial compliance and has been shown to predict cardiovascular events and all-cause mortality, underscoring the clinical importance of improving PWV in delaying hypertension-related cardiovascular complications (Zheng et al., 2024).

In this study, two randomized controlled trials evaluating the effects of aquatic exercise on PWV were included. The pooled analysis showed no statistically significant difference in PWV between the aquatic exercise and control groups (P > 0.05). This finding is consistent with results from land-based exercise interventions in hypertensive patients, where improvements in vascular function and arterial stiffness were also limited over the short term (Ferrier et al., 2001; Ho et al., 2024). Possible explanations include insufficient intervention duration, heterogeneity in baseline arterial stiffness, and differences in measurement techniques.

Although the combined results of the two trials did not reach statistical significance, some individual studies have suggested that aquatic exercise may confer potential benefits for arterial elasticity. However, given the small number of available trials and variability in study design and intervention protocols, definitive conclusions cannot yet be drawn. Additional high-quality, large-sample, long-term randomized controlled trials are needed to further clarify the effects and mechanisms of aquatic exercise on PWV in hypertensive populations.

### 4.1 Study limitations

The overall heterogeneity among the included studies was relatively low (as reflected by low I² values), indicating reasonably consistent pooled effect estimates. Nonetheless, several potential limitations should be considered when interpreting specific outcomes. Variations in aquatic exercise type (e.g., aerobic, resistance, high-intensity interval training), intervention intensity and duration, water temperature and depth, as well as differences in age distribution and baseline blood pressure levels of participants, may all influence individual responses. In addition, antihypertensive medication use may have influenced blood pressure, heart rate, and vascular outcomes; although studies were included only when concomitant treatments were comparable between intervention and control groups, differences in medication type, dosage, or medication changes during follow-up were not consistently reported and could not be further examined. Moreover, methodological differences across studies—such as timing of blood pressure measurements (post-exercise vs resting), PWV assessment techniques, and the accuracy of heart rate monitoring devices—may have affected the estimated effect sizes. Moreover, not all trials clearly reported the exact interval between the last exercise session and the post-intervention assessment; although outcomes were extracted as end-of-intervention resting measures, this reporting variability may have contributed to between-study differences. In addition, most included studies were conducted in Brazil and America, which may limit the generalizability of the findings to hypertensive populations from other regions.

For certain outcomes, such as PWV, only two studies were included and no statistically significant effect was observed, which limits the ability to fully interpret the impact of aquatic exercise on arterial stiffness. Similarly, although heart rate tended to decrease, differences in water temperature settings in some studies may have contributed to inconsistent directions of effect.

Therefore, future research should standardize intervention protocols and the reporting of exercise intensity, water temperature, water depth, and participant characteristics, while enlarging sample sizes and improving trial quality. This would allow more reliable subgroup analyses and help clarify whether the effects of aquatic exercise differ across intervention and patient characteristics.

### 4.2 Future perspectives

The findings of this meta-analysis suggest that aquatic exercise may be associated with favorable cardiovascular-related changes in patients with hypertension, particularly through significant reductions in SBP and DBP, supporting its role as an effective non-pharmacological treatment option. Compared with traditional land-based exercise, aquatic exercise provides a safer and more comfortable environment with reduced joint loading, making it especially suitable for hypertensive patients with joint limitations, higher body weight, or poor exercise adherence. However, current evidence regarding the impact of aquatic exercise on PWV remains limited, and the small number of available studies suggests that short-term aquatic interventions may have only modest effects on arterial elasticity. Consequently, future studies should aim to standardize intervention protocols, increase sample sizes, prolong intervention durations, and adopt rigorously designed randomized controlled trials. In addition, although aquatic exercise-associated changes in SBP and DBP were observed in the present analysis, the comparative effectiveness of different intensities and formats of aquatic exercise on blood pressure and vascular function has not been fully established. With a larger evidence base, future meta-analyses should consider prespecified subgroup analyses (e.g., by water temperature, exercise modality, intensity, and intervention duration) to better explain heterogeneity and identify the most effective aquatic exercise prescriptions for specific patient subgroups. Future research may employ network meta-analyses or head-to-head randomized trials to systematically compare various intensities and forms of aquatic exercise with respect to their effects on blood pressure, PWV, heart rate, and other key cardiovascular indicators, and to further elucidate the underlying physiological mechanisms.

## 5 Conclusion

This meta-analysis suggests that aquatic exercise is associated with favorable changes in key blood pressure indicators in patients with hypertension. Specifically, aquatic exercise was associated with statistically significant changes in SBP and DBP, which may be linked to the unique physiological stimuli of the water environment, including hydrostatic pressure–induced enhancement of venous return, buoyancy-related reductions in cardiac load, and other potential mechanisms proposed in prior literature (e.g., improved endothelial function, decreased sympathetic activity, and increased nitric oxide bioavailability), although these pathways were not directly assessed in the included trials. The findings also indicate that aquatic exercise showed a small but non-significant association with resting heart rate changes in hypertensive patients, and environmental factors such as water temperature may influence heart rate responses. In contrast, current evidence regarding the impact of aquatic exercise on arterial stiffness, as assessed by PWV, remains inconclusive. The pooled analysis did not reveal a statistically significant aquatic exercise-associated change, suggesting that short-term interventions may have limited influence on arterial stiffness. Future studies should expand sample sizes, extend intervention durations, and more rigorously control environmental variables in exercise prescriptions to comprehensively evaluate the long-term effects of aquatic exercise on arterial elasticity and overall cardiovascular health.
