# Supplementary material for: Effect of aquatic exercise on blood pressure and related physiological indicators in hypertensive patients: a systematic meta-analysis of randomized controlled trials
Source: Front Physiol. 2026 Jul 14;17:1876479. doi: 10.3389/fphys.2026.1876479 (PMC13407085; doi:10.3389/fphys.2026.1876479)
Supplement: Supplementary file 1 [file Supplementaryfile1.docx]

Table 1 Search Strategy for the Cochrane Library

| Search | Query | Results |
| --- | --- | --- |
| #1 | MeSH descriptor: [Hypertension] explode all trees | 25492 |
| #2 | (Blood Pressure, High OR Blood Pressures, High OR High Blood Pressure OR High Blood Pressures):ti,ab,kw | 34338 |
| #3 | #1 OR #2 | 55272 |
| #4 | MeSH descriptor: [Water Sports] explode all trees | 759 |
| #5 | (Sports, Water OR Sport, Water OR Water Sport OR Kayaking OR Canoeing OR Water Polo OR Polo, Water OR Surfboarding OR Boating OR Wave Surfing OR Surfing, Wave OR Rowing OR Water Skiing OR Skiing, Water):ti,ab,kw | 3611 |
| #6 | #4 OR #5 | 4244 |
| #7 | #3 AND #6 | 168 |

Table 2 Search Strategy for the Embase

| Search | Query | Results |
| --- | --- | --- |
| #1 | 'hypertension'/exp OR 'hypertension' | 1,559,854 |
| #2 | 'blood pressure, high':ab,ti OR 'blood pressures, high':ab,ti OR 'high blood pressure':ab,ti OR 'high blood pressures':ab,ti | 32,936 |
| #3 | #1 OR #2 | 1,564,005 |
| #4 | 'water sport'/exp OR 'water sport' | 44,160 |
| #5 | 'water sports':ab,ti OR 'sports, water':ab,ti OR 'sport, water':ab,ti OR 'kayaking':ab,ti OR 'canoeing':ab,ti OR 'water polo':ab,ti OR 'polo, water':ab,ti OR 'surfboarding':ab,ti OR 'boating':ab,ti OR 'wave surfing':ab,ti OR 'surfing, wave':ab,ti OR 'rowing':ab,ti OR 'water skiing':ab,ti OR 'skiing, water':ab,ti | 4,238 |
| #6 | #4 OR #5 | 46,356 |
| #7 | #3 AND #6 | 1,021 |

Table 3 Search Strategy for the Web of Science

| Search | Query | Results |
| --- | --- | --- |
| #1 | **Hypertension (Topic) or ''Blood Pressure, High'' (Topic) or ''Blood Pressures, High'' (Topic) or ''High Blood Pressure'' (Topic) or ''High Blood Pressures'' (Topic)** | 689,331 |
| #2 | ''Water Sports'' (Topic) or ''Sports, Water'' (Topic) or ''Sport, Water'' (Topic) or ''Water Sport'' (Topic) or Kayaking (Topic) or Canoeing (Topic) or ''Water Polo'' (Topic) or ''Polo, Water'' (Topic) or Surfboarding (Topic) or Boating (Topic) or ''Wave Surfing'' (Topic) or ''Surfing, Wave'' (Topic) or Rowing (Topic) or ''Water Skiing'' (Topic) or ''Skiing, Water'' (Topic) | 160,874 |
| #3 | #1 AND #2 and Preprint Citation Index (Exclude – Database) | 572 |

Table 4 Search Strategy for the Pubmed

| Search | Query | Results |
| --- | --- | --- |
| #1 | "Hypertension"[Mesh] Sort by: Most Recent | 334162 |
| #2 | (((Blood Pressure, High[Title/Abstract]) OR (Blood Pressures, High[Title/Abstract])) OR (High Blood Pressure[Title/Abstract])) OR (High Blood Pressures[Title/Abstract]) | 22676 |
| #3 | ("Hypertension"[Mesh]) OR ((((Blood Pressure, High[Title/Abstract]) OR (Blood Pressures, High[Title/Abstract])) OR (High Blood Pressure[Title/Abstract])) OR (High Blood Pressures[Title/Abstract])) | 344107 |
| #4 | "Water Sports"[Mesh] Sort by: Most Recent | 29150 |
| #5 | (((((((((((((Sports, Water[Title/Abstract]) OR (Sport, Water[Title/Abstract])) OR (Water Sport[Title/Abstract])) OR (Kayaking[Title/Abstract])) OR (Canoeing[Title/Abstract])) OR (Water Polo[Title/Abstract])) OR (Polo, Water[Title/Abstract])) OR (Surfboarding[Title/Abstract])) OR (Boating[Title/Abstract])) OR (Wave Surfing[Title/Abstract])) OR (Surfing, Wave[Title/Abstract])) OR (Rowing[Title/Abstract])) OR (Water Skiing[Title/Abstract])) OR (Skiing, Water[Title/Abstract]) | 17593 |
| #6 | ("Water Sports"[Mesh]) OR ((((((((((((((Sports, Water[Title/Abstract]) OR (Sport, Water[Title/Abstract])) OR (Water Sport[Title/Abstract])) OR (Kayaking[Title/Abstract])) OR (Canoeing[Title/Abstract])) OR (Water Polo[Title/Abstract])) OR (Polo, Water[Title/Abstract])) OR (Surfboarding[Title/Abstract])) OR (Boating[Title/Abstract])) OR (Wave Surfing[Title/Abstract])) OR (Surfing, Wave[Title/Abstract])) OR (Rowing[Title/Abstract])) OR (Water Skiing[Title/Abstract])) OR (Skiing, Water[Title/Abstract])) | 40650 |
| #7 | (("Hypertension"[Mesh]) OR ((((Blood Pressure, High[Title/Abstract]) OR (Blood Pressures, High[Title/Abstract])) OR (High Blood Pressure[Title/Abstract])) OR (High Blood Pressures[Title/Abstract]))) AND (("Water Sports"[Mesh]) OR ((((((((((((((Sports, Water[Title/Abstract]) OR (Sport, Water[Title/Abstract])) OR (Water Sport[Title/Abstract])) OR (Kayaking[Title/Abstract])) OR (Canoeing[Title/Abstract])) OR (Water Polo[Title/Abstract])) OR (Polo, Water[Title/Abstract])) OR (Surfboarding[Title/Abstract])) OR (Boating[Title/Abstract])) OR (Wave Surfing[Title/Abstract])) OR (Surfing, Wave[Title/Abstract])) OR (Rowing[Title/Abstract])) OR (Water Skiing[Title/Abstract])) OR (Skiing, Water[Title/Abstract]))) | 219 |
